# Plasma D‐dimer and interleukin‐6 are associated with treatment response and progression‐free survival in advanced NSCLC patients on anti‐PD‐1 therapy

**DOI:** 10.1002/cam4.6222

**Published:** 2023-06-16

**Authors:** Chong Chen, Huaru Yin, Yu Zhang, Huan Chen, Jie Xu, Li Ren

**Affiliations:** ^1^ Department of Clinical Laboratory, Tianjin Medical University Cancer Institute and Hospital, National Clinical Research Center for Cancer, Tianjin's Clinical Research Center for Cancer Key Laboratory of Cancer Immunology and Biotherapy, Tianjin Tianjin China; ^2^ Department of Senior Ward, Tianjin Medical University Cancer Institute and Hospital, National Clinical Research Center for Cancer, Tianjin's Clinical Research Center for Cancer Tianjin Key Laboratory of Cancer Prevention and Therapy Tianjin China

**Keywords:** D‐dimer, IL‐6, NSCLC

## Abstract

**Background/Aims:**

Response to therapy after using immune checkpoint inhibitors (ICIs) is unpredictable due to significant interindividual variation in efficacy among advanced non‐small cell lung cancer (NSCLC) patients. The current study centered on the identification of perivascular blood biomarkers for predicting the effectiveness of anti‐programmed cell death protein 1 (anti‐PD‐1) treatment and progression‐free survival (PFS) in advanced NSCLC patients, that could be applied to help determine how to change treatment plans therapeutic regimens for optimizing clinical benefits.

**Methods:**

A comprehensive review of 100 advanced or recurrent NSCLC patients receiving anti‐PD‐1 therapy (Camrelizumab, pembrolizumab, sintilimab, or nivolumab) was conducted between January 2018 and April 2021 in Tianjin Medical University Cancer Hospital. The cutoff values of D‐dimer were selected from rom our previous study, and interleukin‐6 (IL‐6) was divided according to the median. Using computed tomography, tumor response was evaluated in accordance with the Response Assessment Criteria in Solid Tumors, version 1.1.

**Results:**

High IL‐6 level in advanced NSCLC patients was predictive of low efficacy and a short PFS duration after anti‐PD‐1 therapy. An increased D‐dimer value of 981 ng/mL was significantly predictive of disease progression in NSCLC patients treated with anti‐PD‐1 and high D‐dimer expression predictive of short duration of PFS. Further studies on the correlation between IL‐6, D‐dimer, and anti‐PD‐1 efficacy in NSCLC patients stratified by gender revealed that D‐dimer and IL‐6 levels were significantly associated with the risk of PFS in male patients.

**Conclusions:**

High IL‐6 content in peripheral blood in patients with advanced non‐small cell lung cancer may contribute to poor anti‐PD‐1 efficacy and short duration of PFS through inducing alterations in the tumor microenvironment. D‐dimer in peripheral blood is predictive of hyperfibrinolysis and contributes to the release of tumor‐driven specific factors, leading to poor effects of anti‐PD‐1 therapy.

## 1 INTRODUCTION

Lung cancer, the foremost reason of tumor‐related mortality globally, is clinically and pathologically distinguished into two subtypes: NSCLC, constituting 80% cases and SCLC, constituting 20% cases.^1^, ^2^ The majority of NSCLC patients are diagnosed at III or IV stages and therapeutic alternatives remain primarily limited to cytotoxic chemotherapy. In regard to tyrosine kinase inhibitors (TKIs), one of the possibilities includes epidermal growth factor receptors (EGFR) wild‐type allele amplification, highlighting the importance of EFGR genotype for the treatment efficacy.^3^ Molecularly targeted drugs such as EGFR and TKIs are effective in patients with EGFR or ALK mutations in lung cancer.^4^, ^5^ Recent advancements in immune checkpoint inhibitors (ICIs) for the management of cancer have heightened therapeutic expectations for NSCLC patients.^6^, ^7^, ^8^, ^9^ Unlike traditional treatments (chemotherapy, radiotherapy, or targeted therapies), which mainly target the tumor cells themselves, immunotherapy directly improves or restores tumor surveillance and depleted host antitumor immune responses.^10^, ^11^, ^12^ The successful application of immune checkpoint inhibitors of anti‐PD‐1 and PD‐L1 (programmed cell death protein ligand‐1) has attracted considerable interest in the field of immuno‐oncology for a variety of advanced cancers.^13^, ^14^, ^15^ PD‐L1 blockade also applies in other treatments based on the theories of immunotherapy. For example, encouraging results have been achieved with two antibodies against PD‐1 (nivolumab and pembrolizumab) and PD‐L1 (atezolizumab and durvalumab), which have recently been approved by the European Medicines Agency (EMA) and the US Food and Drug Administration (FDA) for treating advanced NSCLC.^16^, ^17^, ^18^, ^19^

Notwithstanding the escalating incidence of pulmonary carcinoma and its frequent detection at advanced stages of progression, treatment options for this disease is consistently ameliorating. As subsequent treatment with anti‐PD‐1 has been ameliorated, identification of biomarkers with utility in predicting response to therapy is critical to facilitate the accurate selection of patients who are most likely to benefit from this intervention. Effective conservative treatment options are available for advanced lung cancer patients to avoid unnecessary costs and time‐wastage, as well as risk of immune‐related adverse events (irAEs) associated with immunotherapy. PD‐L1 expression in tumor tissues has been established as a reliable biomarker for prediction of response to ICI treatment. Other predictive biomarkers of ICI efficacy in advanced NSCLC include tumor mutation burden (TMB) in tumor tissue and blood, microsatellite instability (MSI), and DNA damage repair (DDR) machinery. However, these tests are not only time‐consuming but also challenging to implement in daily clinical practice.^20^, ^21^ Precise serum markers with significant predictive value in treatment response and progression‐free survival (PFS) remain to be determined.

Hemostatic factors are closely related to tumor progression.^22^ Malignancy may trigger activation of the clotting cascade, which generates thrombin and fibrin and stimulates procoagulant features of platelets, leukocytes, and endothelial cells. Soluble D‐dimer, produced as a result of fibrin network degradation from fibrin‐bound plasmin, is a pivotal biomarker of hypercoagulability and thrombosis associated with clinical indications, such as tumor stage, metastasis, and growth, in a large number of cancers, and used to predict survival independent of clinical stage, histologic tumor type, and performance status in lung cancer patients.^23^, ^24^ Even in cases of a clear correlation between D‐dimer levels and cancer progression, the issue of whether D‐dimer can be effectively used to predict response of anti‐PD‐1 therapy remains to be clarified. In a previous study by our group, improving the cutoff value of D‐dimer to 981 ng/mL had utility in predicting poor prognosis in advanced NSCLC patients.^25^

Accumulating evidence suggests that inflammatory signals from tumor cells and the surrounding microenvironment, such us IL‐6, are strongly associated with promote tumor growth in several cancer types.^26^ Activation of the IL‐6/STAT‐3 signaling axis is reported to stimulate tumorigenesis.^27^ Various cytokines are produced by pro‐inflammatory mediators from the surrounding tumor tissue to suppress immune response, so that pro‐inflammatory mediators can modulate therapeutic effect.^28^ In addition, IL‐6 may promote drug resistance associated with increased expression of the multidrug resistance gene, mdr1, and upregulation of C/EBPβ and C/EBPδ (the CCAAT enhancer‐binding protein family of transcription factors).^29^ Since IL‐6 can be utilized as an indicator for predicting the efficacy of anti‐PD‐1 therapy, the potential correlation between these factors was further explored. The association is also between high IL‐6 with negative effects of therapy in other tumors.^30^

The associations of particular serum blood biomarkers used in routine clinical practice with treatment response and PFS of patients with advanced NSCLC on anti‐PD‐1 therapy were investigated, with the aim of identifying available biomarkers that could aid in predicting treatment responses or PFS with clinical applicability. Our results suggest that the D‐dimer and IL‐6 have significant value in predicting response to anti‐PD‐1 therapy.

## 2 METHODS

### 2.1 Patients and samples

We enrolled 100 patients receiving treatment with anti‐PD‐1 (nivolumab, sintilimab, or pembrolizumab) for advanced or recurrent NSCLC in a retrospective study between January 2018 and February 2020 in Tianjin Medical University Cancer Institute and Hospital. Nivolumab was administered intravenously to patients at a dosage of 3 mg/kg every 2 weeks. Other patients got 200 mg of sintilimab, 200 mg of camrelizumab, or 200 mg of pembrolizumab intravenously every 3 weeks. Clinicopathological characteristics of patients were noted, including age, at which therapy was initiated, gender, Eastern Cooperative Oncology Group Performance Status (ECOG PS), line of therapy, history of smoking, history of radiotherapy, clinical stage (according to the 7th edition guidelines of the International Association for the Study of Lung Cancer), driver oncogene mutation status, and histology. Levels of plasma D‐dimer and serum inflammatory marker IL‐6 were measured when anti‐PD‐1 therapy was started. Every 6 to 8 weeks, a chest computed tomography (CT) was utilized to assess the tumor response in accordance with the Response Evaluation Criteria in Solid Tumors, version 1.1.^31^ Information on patient's clinical and follow‐up care was collected from their medical files. The time for follow‐up ended on November 30, 2020.

### 2.2 PD‐L1 tumor expression and driver oncogene mutation analysis

Data regarding the expression status of PD‐L1 tumors and the mutational status of driver oncogenes, including epidermal growth factor receptor (EGFR) and anaplastic lymphoma kinase (ALK), were extracted from the medical records of the patients. Immunohistochemical staining was performed using PD‐L1 antibody clone 28–8 c (1:300; Abcam, rabbit IgG) following the manufacturer instructions.^32^ The presence of EGFR gene mutations in tumor samples was identified via reverse transcription polymerase chain reaction (RT‐PCR), and ALK status in tumor tissue assessed using fluorescence in situ hybridization (FISH).^33^ The clinical status of patients was kept a secret from two experienced technicians who reviewed the PD‐L1 date. A consensus was reached for the final assessment, and tumor proportional score (TPS) was employed to categorize PD‐L1 tumor expression.

### 2.3 Confirmation of the cutoff value for D‐dimer

In a previous study by our group, prognosis of NSCLC patients was effectively improved by optimization of the cutoff value for D‐dimer.^25^ In the previous study, retrospective analysis was done on 233 patients with advanced NSCLC. We selected to increase the plasma D‐dimer cutoff value to 981 ng/mL instead of the original 500 ng/mL which was commonly used in clinical practice. The Cox proportional hazard regression model was employed for multivariable analysis, alongside univariable analysis utilizing the Kaplan–Meier method and log‐rank test. The high D‐dimer content serves as an effective predictor of poor prognosis in advanced NSCLC cases. Accordingly, the optimal cutoff value of 981 ng/mL D‐dimer was applied in this study.

### 2.4 Statistical analysis

Continuous variables were evaluated as median (quartile range) and categorical variables as whole numbers and proportions. The plasma D‐dimer cutoff value was based on a prior study conducted by our team.^25^ Progression‐free survival (PFS) curves for the study population were generated using the Kaplan–Meier method and differences examined with the log‐rank test. Univariable Cox regression analysis was performed to evaluate the association between PFS and other variables. Including all factors with *p* values <0.05 of clinical significance, multivariable Cox regression with backward selection was further performed to identify the independent factors predictive of PFS. Variables of clinical significance associated with PFS were assessed a priori based on clinical importance, scientific knowledge, and previously identified predictors. In addition, three‐knotted restricted cubic spline (RCS) regression was employed to determine the possible nonlinear dependency of the relationship between PFS and plasma indicators (IL‐6 and D‐dimer). Multivariable Cox regression analysis stratified by sex was additionally performed. Statistical analyses were conducted using SPSS (version 26.0) and R (version 4.0.3). All tests were two‐sided and data considered statistically significant at *p* values <0.05.

## 3 RESULTS

**FIGURE 1:**
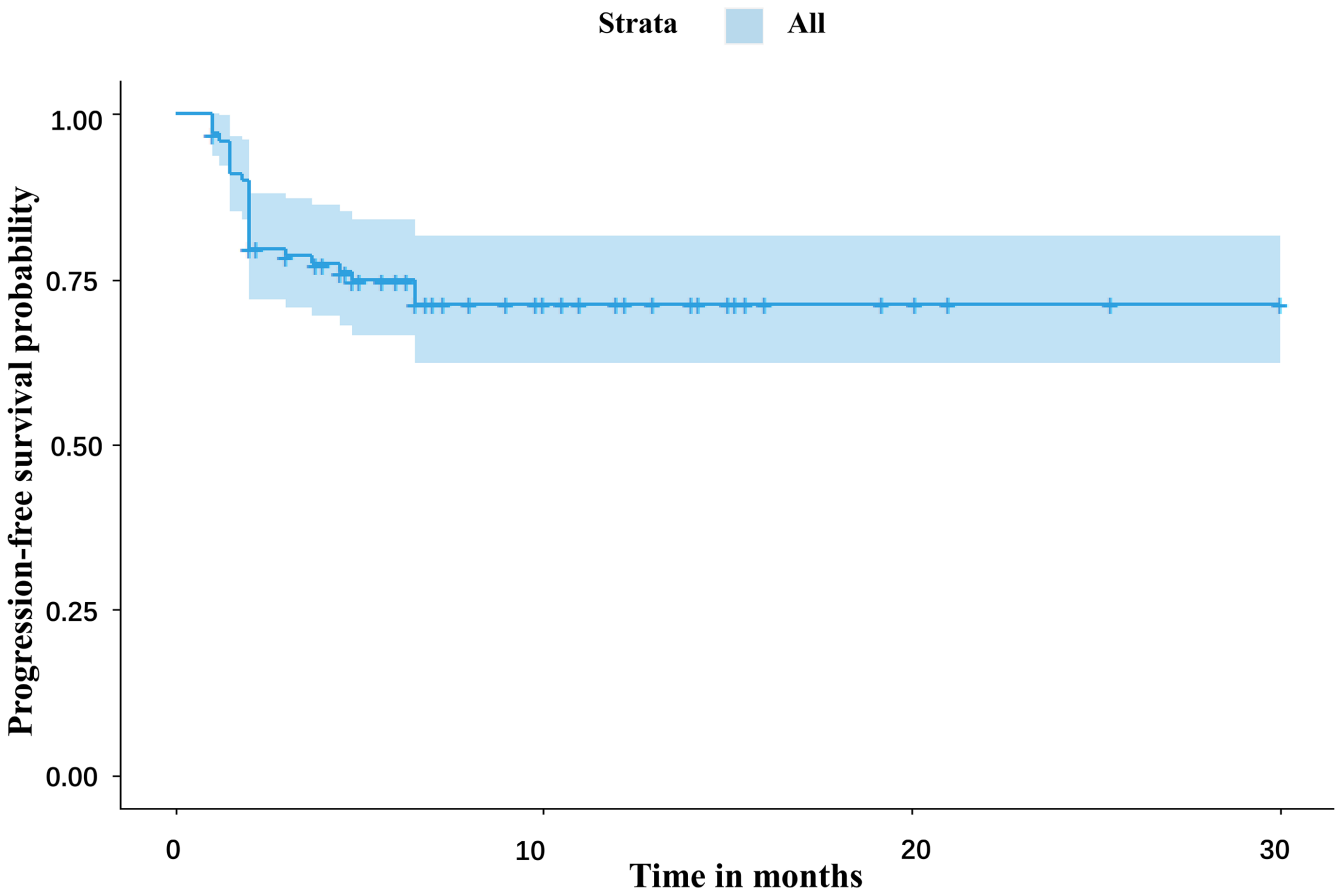
Progression‐free survival of enrolled patients.

The basic characteristics of NSCLC patients are shown in Table 1. In the high IL‐6 group, median age of patients was 63.5 years (IQR, 54–66 years) and 86% (43 of 50) were male. The ECOG performance scores of most patients is 0 and 1 (45 of 50), and a high proportion were diagnosed with stage IV disease (46 of 50, with 10 at stage IVA and 36 at stage IVB). Overall, 82% patients (41 of 50) were current or former smokers. Adenocarcinoma and squamous cell carcinoma accounted for 38% and 42% of pathological lung tumors, respectively. The drugs used for treatment included nivolumab (7 cases), pembrolizumab (20 cases), sintilimab (19 cases), and camrelizumab (4 cases), respectively. The median levels of preoperative D‐dimer and IL‐6 were determined as 1560.35 ng/mL and 10.24 pg/mL, respectively. In the high D‐dimer group, median age of patients was 64 years (IQR, 54.5–68 years), and 78% (39 of 50) were male. The ECOG performance scores of most patients is 0 and 1 (42 of 50) and a high proportion were diagnosed with stage IV disease (43 of 50, with 12 at stage IVA and 31 at stage IVB). Overall, 82% patients (35 of 50) were current or former smokers. Adenocarcinoma and squamous cell carcinoma accounted for 32% and 42% of pathological lung tumors, respectively. The drugs used for treatment included nivolumab (8 cases), pembrolizumab (19 cases), sintilimab (16 cases), and camrelizumab (2 cases), respectively. The median levels of preoperative D‐dimer and IL‐6 were determined as 1698.85 ng/mL and 10.36 pg/mL, respectively. The abnormal proportion of preoperative D‐dimer values was 45% based on the cutoff value determined in our previous study. We observed 26 (26%) cases of disease progression. The medium PFS was 6.0 months (Figure 1).

**TABLE 1:** Demographics and clinicopathologic characteristics of patients in the four groups including the low IL‐6 level, the high IL‐6 level, the low D‐dimer level, and the high IL‐6 level.

	IL‐6 (pg/mL)		D‐Dimer (ng/mL)	
	<6.37 (low)	≥6.37 (high)	<981 (low)	≥981 (high)
Sex				
Male	41 (41)	43 (43)	45 (45)	39 (39)
Female	9 (9)	7 (7)	10 (10)	6 (6)
Age (years)	64 (58.755–68.25)	63.5 (54–66)	63 (57–68)	64 (54.5–68)
ECOG PS				
0	9 (9)	5 (5)	9 (9)	5 (5)
1	36 (36)	40 (40)	39 (39)	37 (37)
2	4 (4)	5 (5)	6 (6)	3 (3)
3	1 (1)	0 (0)	1 (1)	0
Line of treatment				
First	18 (18.2)	15 (15.2)	18 (18.2)	15 (15.2)
Second	21 (21.2)	21 (21.2)	24 (24.2)	18 (18.2)
Third or higher	10 (10.1)	14 (14.1)	12 (12.2)	12 (12)
Smoking history				
Current smoker	19 (19)	18 (18)	10 (20)	17 (17)
Ex‐smoker	21 (21)	23 (23)	26 (26)	18 (18)
Never smoker	10 (10)	9 (9)	9 (9)	10 (10)
Clinical stage				
IIIB	4 (4)	4 (4)	6 (6)	2 (2)
IVA	11 (11)	10 (10)	9 (9)	12 (12)
IVB	35 (35)	36 (36)	40 (40)	71 (71)
Mutation status (EGFR or ALK)				
Wild‐type	41 (47.7)	37 (43)	45 (52.3)	33 (38.4)
Mutant‐type	1 (1.2)	7 (8.1)	2 (2.3)	6 (7)
Histology				
Adenocarcinoma	22 (22)	19 (19)	25 (25)	16 (16)
Squamous cell carcinoma	18 (18)	21 (21)	18 (18)	21 (21)
Others or unknown	10 (10)	10 (10)	12 (12)	8 (8)
Immune checkpoint inhibitors				
Nivolumab	6 (6)	7 (7)	5 (5)	8 (8)
Pembrolizumab	19 (19)	20 (20)	20 (20)	19 (19)
Sintilimab	21 (21)	19 (19)	24 (24)	16 (16)
Camrelizumab	4 (4)	4 (4)	6 (6)	2 (2)
BMI (kg/m^2^)	24.10 (20.76–25.98)	24.43 (21.71–25.93)	24.38 (21.05–25.71)	24.26 (21.59–25.95)
D‐dimer (ng/mL)	676.71 (398.26–907.59)	1560.35 (974.73–2350.75)	575.01 (377.69–815.76)	1698.85 (1460.78–2817.78)
IL‐6 (pg/mL)	4.12 (2.10–5.12)	10.24 (7.80–19.23)	4.64 (3.02–6.34)	10.36 (6.58–20.41)

In Table 2, model 1 listed the results of univariable analysis. PFS was significantly associated with IL‐6 (*p* < 0.001), D‐dimer (*p* < 0.001), high IL‐6 (*p* = 0.001), and high D‐dimer (*p* < 0.001) levels. In model 2, the factors that is basic information (e.g., age and sex) and lifestyle (e.g., smoking, BMI, and ECOG PS) of patients were included in multivariable analysis. In the multivariable Cox regression model, IL‐6 (*p* < 0.001), D‐dimer(*p* < 0.001), high IL‐6 (*p* = 0.001) value, and high D‐dimer (*p* < 0.001) value were closely related to PFS. Subsequently, we added the clinical factors (e.g., line of treatment, clinical stage, mutation status, histology, and immune checkpoint inhibitors) into model 3 based in model 2. IL‐6 (*p* = 0.002), D‐dimer(p = 0.017), high IL‐6 (p = 0.004) value, and high D‐dimer (*p* < 0.001) value were also closely related to PFS.

**TABLE 2:** Univariable and multivariable Cox regression analysis of associations of variables with PFS.

		IL‐6 (pg/mL)		D‐dimer (ng/mL)		IL‐6	D‐dimer
		<6.37	≥6.37	<981	≥981		
Model 1	HR (95% CI)	1	6.277 (2.152–18.304)	1	7.369 (2.525–21.506)	1.055 (1.033–1.078)	1
	*p*		0.001		<0.001	<0.001	<0.001
Model 2	HR (95% CI)	1	7.835 (2.444–25.125)	1	7.349 (2.476–21.816)	1.064 (1.035–1.093)	1 (1–1.001)
	*p*		0.001		<0.001	<0.001	<0.001
Model 3	HR (95% CI)	1	5.624 (1.714–18.455)	1	14.088 (3.262–60.844)	1.076 (1.026–1.127)	1 (1–1.001)
	*p*		0.004		<0.001	0.002	0.017

**FIGURE 2:**
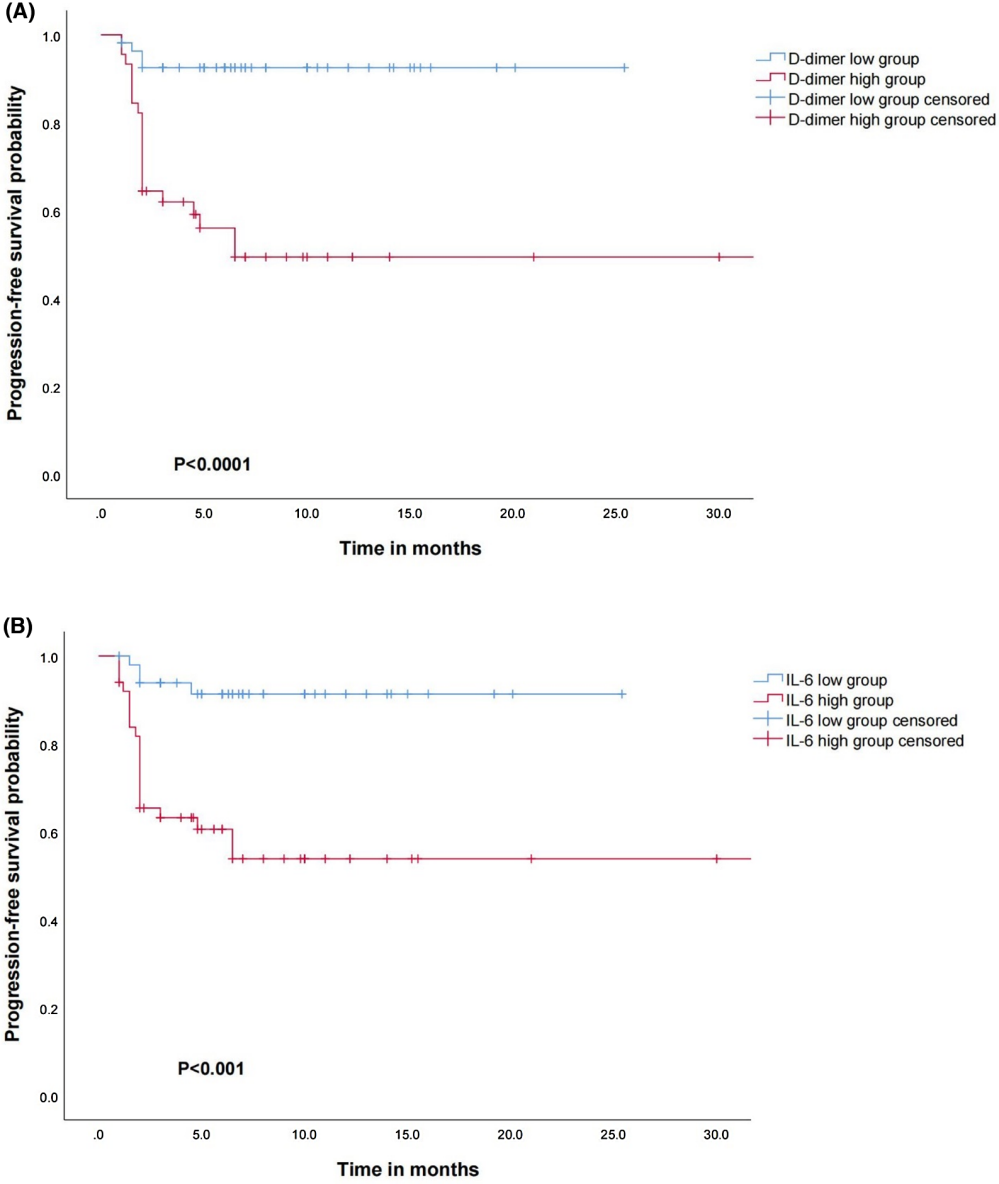
(A, B) Comparison of PFS rates of patients classified into high and low IL‐6 and D‐dimer groups.

To further assess the predictive value of IL‐6 and D‐dimer levels, the probability of PFS was plotted as Kaplan–Meier curves. IL‐6 was categorized into two groups (by median) and D‐dimer into two groups (by cutoff) based on previous findings (Figure 2). Patients with the highest preoperative IL‐6 and D‐dimer levels had substantially poorer PFS compared with those with the lowest levels (*p* < 0.001).

**FIGURE 3:**
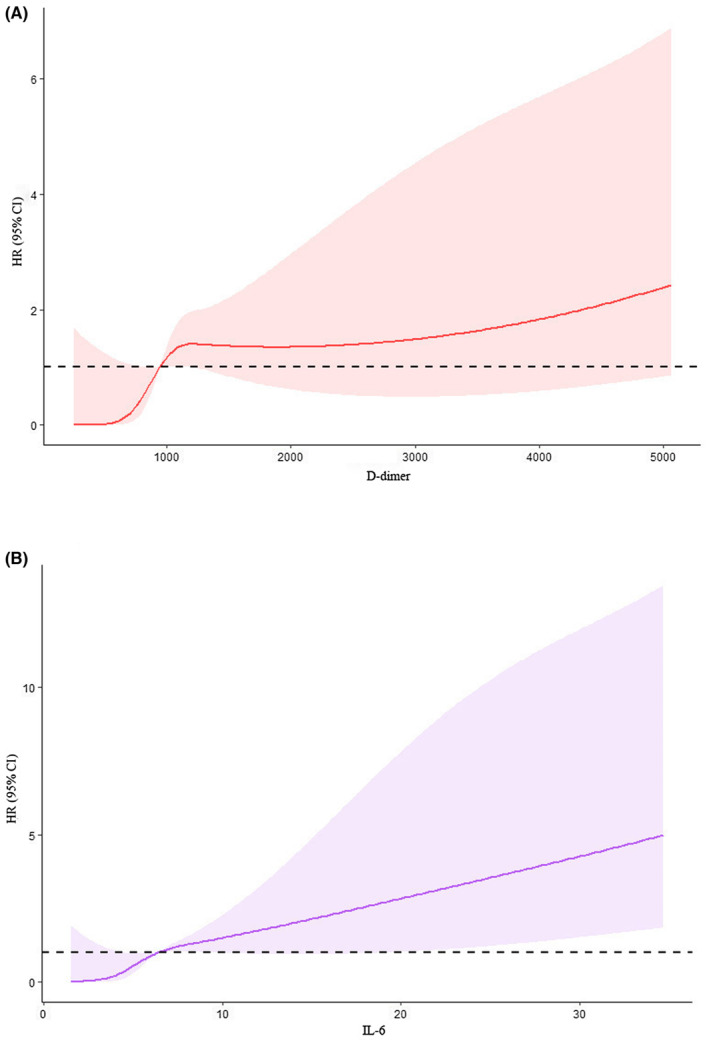
(A, B) Linear association between (A) D‐dimer or (B) IL‐6 and risk of PFS determined using restricted cubic spline regression by univariable model.

Considering IL‐6 and D‐dimer as continuous variables, a linear association between these factors and risk of PFS using restricted cubic spline regression was observed (for nonlinearity, *p* = 0.108 for IL‐6, *p* = 0.184 for D‐dimer) (Figure 3).

The correlation between the cancer progression with gender was studied in previous study, the correlations among IL‐6, D‐dimer, and PD‐1 efficacy in lung cancer patients of different genders were further investigated. In multivariable analysis, the factors that is basic information (e.g., age) and lifestyle (e.g., smoking, BMI, and ECOG PS) of patients were included. Conclusionally, the D‐dimer (HR = 1, 95% CI: 1.000–1.001, *p* < 0.001) and IL‐6 (HR = 1.06, 95% CI: 1.029–1.091, *p* < 0.001) level was prominently associated with PFS in the male patients. By contrast, no significant relationship between the D‐dimer (HR = 1, 006% CI: 0.987–1.026, *p* < 0.508) or IL‐6 level (HR = 1.312, 95% CI: 0.92–18.644, *p* = 0.814) and PFS was fund in female patients (Table 3).

**TABLE 3:** Multivariable Cox regression analysis stratified by sex.

Factors	Female		Male	
	HR (95% CI)	p	HR (95% CI)	p
IL‐6	1.312 (0.092–18.644)	0.841	1.06 (1.029–1.091)	<0.001
D‐dimer	1.006 (0.987–1.026)	0.508	1 (1–1.001)	<0.001

## 4 DISCUSSION

Unprecedented clinical benefits for advanced NSCLC patients have been achieved using immune checkpoint inhibitors, with clinically established efficacy based on PD‐L1 expression in tumor cells. However, the efficacy of anti‐PD‐1 treatment against lung cancer is significantly different among patient groups and irAEs may commonly occur after immunotherapy. PD‐L1 expression cannot be accurately used to predict survival leads to inadequate benefits from ICI therapy. Considering the increasing number of patients on anti‐PD‐1 therapy, high expenditure of treatment, and large subset of NSCLC patients in China, the identification of predictive biomarkers that may be used to select suitable patient populations likely to significantly benefit from ICI therapy remains a major challenge.

Using the new D‐dimer cutoff value obtained from a previous study, we demonstrated its utility as a predictive biomarker of the anti‐PD‐1 treatment response based on the relationship between D‐dimer and PFS established through Cox regression analysis. Notably, increasing the cutoff value of D‐dimer to 981 ng/mL was effective in predicting disease progression in NSCLC patients who were treated by anti‐PD‐1 therapy after 6–8 weeks. IL‐6 also showed significant value in predicting response of NSCLC patients to anti‐PD‐1 therapy. High IL‐6 expression (>6.37 pg/mL) in advanced NSCLC cases was associated with poor treatment response and short PFS following anti‐PD‐1 therapy, which could be potentially attributed to IL‐6‐mediated blockage of the PD‐1/PD‐L1 axis. Further examination of the correlations among IL‐6, D‐dimer, and PD‐1 efficacy in lung cancer patients stratified by gender revealed a significant association of IL‐6 levels with risk of PFS in male but not female patients.

Plasma coagulation, circulating platelets, and the vascular endothelium can regulate hemostasis, this complicated and dynamic process plays a vital role in balancing bleeding and clot formation.^34^ The homeostatic balance between coagulation and bleeding is maintained in normal people, while changes are prevalent in patients with malignant tumors. Malignancy has important function in tumor cell‐associated clot‐promoting properties that trigger activation of the coagulation cascade, generating thrombin and fibrin, in addition to stimulating cellar procoagulant traits of platelets, leukocytes and endothelial cells.^22^ Degradation of the fibrin network is stimulated from fibrin‐bound plasmin into soluble D‐dimer and E fragments. Increased levels of D‐dimer, a key biomarker of coagulation and thrombosis, induce the holistic activation of clotting and fibrinolysis.^35^ Tumor occurrence and progression can be contributed by several of these mechanisms.^36^ Recently, D‐dimer was validated as a clinically applicable prognostic biomarker. The relationship between D‐dimer and cancer progression has been explored in a number of earlier studies^37^, ^38^ showing that patients with elevated D‐dimer contents are more prone to early disease progression. Consistently, previous experiments by our group confirmed a relationship between D‐dimer and lung cancer. The group of Chen further demonstrated the utility of high levels of D‐dimer as a predictive factor of poorer PFS and OS of patients with SCLC. In cases where the D‐dimer content decreased to normal levels after 2 cycles of chemotherapy, survival was longer relative to patients who received 2 cycles of chemotherapy and still possessed high D‐dimer levels.^39^ To date, limited studies have focused on the relationship between D‐dimer content and prediction of response to anti‐PD‐1 therapy in NSCLC patients. However, a significant association of D‐dimer with development of lung cancer has been previously documented. Earlier, our group showed that elevating the D‐dimer cutoff to 981 ng/mL improved prognostic prediction in patients with advanced NSCLC.^25^ In the current study, application of the higher D‐dimer cutoff content to predict response to anti‐PD‐1 treatment in NSCLC patients and assess of disease progression validated the association of high D‐dimer content with short duration of PFS.

The relationship between the inflammatory microenvironment and tumor cells profoundly impacts tumor growth, metastasis, and drug resistance.^40^ Within the microenvironment, tumor cell upgrowth is regulated by a number of pro‐inflammatory cytokines. IL‐6 is a major tumor‐interrelated inflammatory cytokine, whose production and exudation by multiple types of cells involving the tumor cells. This cytokine is deregulated and vital in the progression of human cancer via activating oncogenic pathways. For instance, activation of STAT3 is regulated by IL‐6, which may be relevant to lung tumorigenesis.^41^ STAT3 promotes the expression of downstream genes, such as Bcl2, that control cell proliferation, invasion, and survivability.^42^ Activating the IL‐6/STAT‐3 signaling axis has been identified as a crucial step that regulates various survival signaling pathways to promote tumorigenesis.^27^ A correlation between response to PD‐1 blockade and low IL‐6 levels in bronchoalveolar lavage fluid was discovered in a mouse model of NSCLC by Akbay et al. In patients and peripheral blood is possibly similar with this mechanism.^43^ Kang et al.^44^ reported that the peripheral interleukin‐6 level at baseline is relevant to the clinical efficacy of anti‐PD‐1 therapy in NSCLC patients, further supporting its utility as a predictor of response. In this study, among the three inflammatory markers (NLP, PLR, and IL‐6) selected for analysis as independent predictors of survival and candidate biomarkers of the efficacy of anti‐PD‐1 therapy, only IL‐6 was significantly relevant to efficacy and PFS. Notably, high IL‐6 expression was correlated with poor treatment efficacy in NSCLC patients treated by anti‐PD‐1 therapy. Tiziana et al.^45^ illustrated that there were gender‐based differences in the immune system which in turn lead to significant differences in the response to immunotherapy for advanced lung cancer. Therefore, in order to properly assess cancer response and disease control, the conclusions of this study conducted in female lung cancer patients, considering only age and reproductive status, may not be absolutely applicable to male patients, or conversely.^45^ Previously, elevated IL‐6 was shown to be correlated with the susceptibility and incidence of liver cancer in male mice.^46^ One potential explanation for this finding is that in female mice, IL‐6 generation is obstructed by estrogen steroid hormones, which could protect against cancer progression. Consistent with data obtained using the mouse model, IL‐6 exerted a more significant predictive effect in male than female patients in this study. We also reached conclusion that D‐dimer was more associated with PFS in male than female patients.

In conclusion, high IL‐6 content in peripheral blood in patients with advanced non‐small cell lung cancer may contribute to poor anti‐PD‐1 efficacy and short duration of PFS through inducing alterations in the tumor microenvironment. D‐dimer in peripheral blood is predictive of hyperfibrinolysis and contributes to the release of tumor‐driven specific factors, leading to poor effects of anti‐PD‐1 therapy. The key strength of this study was the adoption of commonly used clinical indications to predict response to anti‐PD‐1 treatment and utility of the new D‐dimer cutoff content based on previous findings. However, a major limitation was the relatively small number of NSCLC patients included for analysis. Further studies on larger populations of advanced NSCLC patients are warranted to validate the risk assessment model.

## AUTHOR CONTRIBUTIONS

Chong Chen, Yu zhang and Ru Hua Yin; study concept and design, acquisition of data, analysis and interpretation of data, and drafting of the manuscript. Huan Chen and Chong Chen; critical revision of the manuscript for important intellectual content. Ru Hua Yin and Jie Xu; acquisition of data; analysis and interpretation of data. Chong Chen, Yu zhang and Li Ren; statistical analysis, technical, or material support. Chong Chen and Li Ren; study concept and design, obtained funding, and study supervision.

## CONFLICT OF INTEREST STATEMENT

The authors declare no potential conflicts of interest.

## ETHICS STATEMENT

This study was carried out under the revised Declaration of Helsinki. The Ethics Committee approved the study of the Tianjin Medical University Cancer Institute & Hospital (NO. EK2021043). Prior to the study, all participants signed an informed consent form.

## Data Availability

We have obtained data from corresponding authors upon reasonable request to support our findings.
